# Co-existence of lung carcinoma metastasis and enchondroma in the femur of a patient with Ollier disease

**DOI:** 10.1007/s00428-020-02936-z

**Published:** 2020-10-13

**Authors:** Xudong Wang, Xiaohui Zhang, Wenling Pan, Yuedong Han, Yanwei Li, Haibin Sun, Pancras C. W. Hogendoorn, Hong Cheng

**Affiliations:** 1 Department of Pathology, The Fifth Affiliated Hospital of Zhengzhou University, Zhengzhou, Henan Province China; 2 Department of Pathology, H. Lee Moffitt Cancer Center and Research Institute, Tampa, FL USA; 3 Department of Radiology, The Fifth Affiliated Hospital of Zhengzhou University, Zhengzhou, Henan Province China; 4 Department of Radiology, GaoXin Hospital, Xi’an Jiao Tong University, No.16, South Tuanjie Road, Xi’an, Shaanxi China; 5 Department of Pathology, Leiden University Medical Center, Leiden, The Netherlands

**Keywords:** Bone tumour, Ollier disease, Tumour-to-tumour metastasis, Lung carcinoma, Enchondroma

## Abstract

Tumour-to-tumour metastasis is very unusual and has been defined as a tumour metastasis into another histologically different tumour. It is extremely rare in bone. We report a case of lung squamous cell carcinoma metastasized to an enchondroma in the femur of a patient with Ollier disease. A 60-year-old female had a history of a poorly differentiated squamous cell carcinoma of the lung. She underwent a video-assisted thoracoscopic lobectomy, and a follow-up MRI scan showed three lesions in the left distal femur and proximal tibia, which were initially interpreted as metastasis on radiology. Resection of the left proximal tibial lesion was performed, and the pathological findings were consistent with enchondroma with no evidence of metastasis. Subsequent curettage of lesions in the distal left femur revealed metastatic poorly differentiated carcinoma with foci of hyaline cartilage, which was most consistent with metastatic carcinoma in a pre-existing enchondroma. The MRI films were re-reviewed. Characteristic MRI features of enchondroma were found in the lesion in the left proximal tibia and one of the lesions in the left distal femur, while the features of the other lesion in the left distal femur included cortical destruction and extensive oedema in surrounding soft tissue, which were consistent with a malignant tumour. In addition, the enchondroma in the lateral condyle showed blurring and irregular inner margin and adjacent bone oedema, which likely represents a co-existing metastatic tumour and enchondroma. The difference in lineage was confirmed by immunohistochemistry. The final diagnosis was metastatic poorly differentiated carcinoma of the lung into a co-existent enchondroma. The diagnosis can be challenging and could be easily overlooked both radiologically and histologically. Thorough clinical and radiological information is critical for the diagnosis, and despite a very unusual event, awareness of the tumour-to-tumour metastasis phenomenon can avoid an inaccurate diagnosis by the pathologist, therefore preventing inappropriate clinical intervention.

## Background

Tumour-to-tumour metastasis is a rare phenomenon that has been described as an occurrence of distant tumour metastasis into another histologically different tumour. The metastatic tumour is usually an aggressive high-grade neoplasm, while the primary tumour is often a more indolent one. Although tumour-to-tumour metastasis cases have been reported intermittently, it is extremely rare in the bone. We herein present a case of a patient with Ollier disease with multiple enchondroma [1] and within an enchondroma in the femur a tumour-to-tumour metastasis from a lung squamous cell carcinoma. To our knowledge, this report is the first case of tumour-to-tumour metastasis from a lung carcinoma to an enchondroma in the context of Ollier disease.

## Case report

**Fig. 1:**
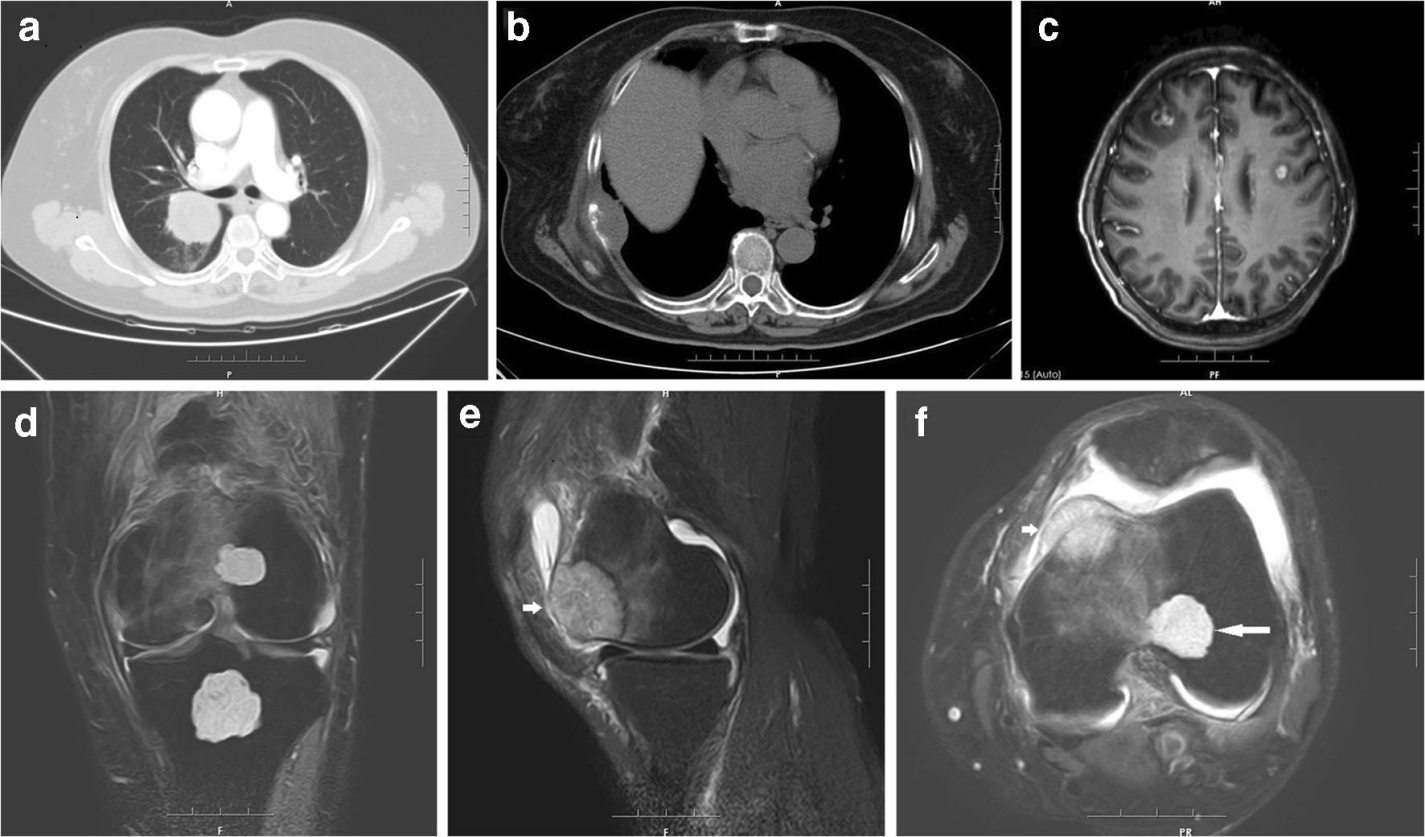
**a** Contrast-enhanced CT scan of the chest revealed a mass with irregular margin in the posterior segment of the right upper lobe of the lung. **b** Osteolytic metastasis in the right 7th rib with soft tissue mass formation. **c** Contrast-enhanced MRI image of the brain showed multiple metastases in the bilateral frontal lobes. **d**–**f** Coronal (**d**), sagittal (**e**) and axial (**f**) fat-suppressed T2-weighted MRI images demonstrated three lesions in the left femur and tibia, including a lesion in the tibia, a lesion in the anterior part of the medial condyle of femur (short arrow, metastatic carcinoma) and a lesion located in the lateral condyle (long arrow, co-existing tumour of metastasis and enchondroma)

**Fig. 2:**
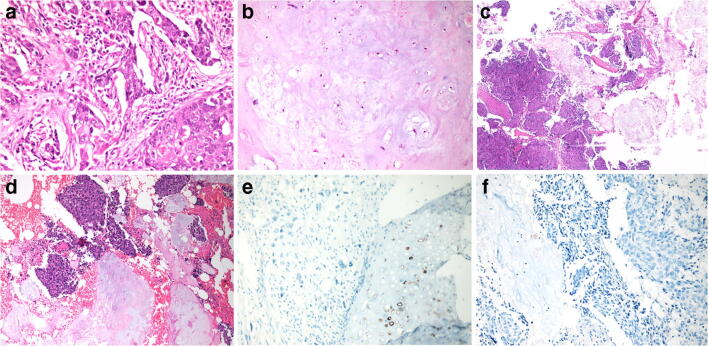
**a** Thoracoscopic lobectomy from the right upper lobe of the lung revealed poorly differentiated squamous cell carcinoma. **b** The tumour from the left proximal tibia was consistent with enchondroma without metastatic component. **c**, **d** Resection from the left distal femur revealed metastatic poorly differentiated carcinoma and foci of hyaline cartilage was present in the metastatic carcinoma, consistent with a metastatic carcinoma in a pre-existing enchondroma. **e** Immunohistochemical stain for IDH1 was positive in the hyaline cartilage cells but negative in the squamous cell carcinoma cells in lung. **f** Immunohistochemical stain for IDH1 was weakly positive in the enchondroma cells but negative in the metastatic squamous cell carcinoma cells

A 60-year-old female was admitted to our hospital, complaining of cough and expectoration for 2 weeks. A contrast-enhanced computed tomography (CT) scan of the chest revealed a mass with an irregular margin in the posterior segment of the right upper lobe of the lung (Fig. 1a). CT-guided percutaneous needle biopsy of the lung was consistent with a diagnosis of poorly differentiated squamous cell carcinoma. The patient underwent a video-assisted thoracoscopic lobectomy. Grossly, the lung lobe measured 16.0 × 12.0 × 5.0 cm, and the cut section reveals a tan tumour. The tumour was located near the hilum of the lung and measured 5.0 × 4.5 × 3.5 cm in dimension. Histological examination revealed a poorly differentiated squamous cell carcinoma with necrosis and brisk mitotic activity (Fig. 2a).

Nearly 4 months after the surgery, the patient was admitted to our hospital for the second time, complaining of swelling, pain and loss of range of movement in the left knee joint. Magnetic resonance imaging (MRI) scan revealed tumours in the left distal femur and the left proximal tibia, which were initially radiologically interpreted as tumour metastasis (Fig. 1d–f). Resection of the left proximal tibial lesion and bone grafting was performed. The pathological diagnosis was enchondroma (Fig. 2b). Since there was a concern of metastatic carcinoma based on radiological findings, curettage of distal left femoral lesion and bone grafting were performed as well. Grossly, the resected specimen was grey-brown fragments measuring 2.2 × 1.5 × 0.8 cm in toto. On microscopic examination, there were sheets of large polygonal malignant cells with pink or clear cytoplasm, vacuolated nuclei and prominent nucleoli. The tumour infiltrated into bony trabeculae with focal haemorrhage and necrosis. The differential diagnosis on morphology was dedifferentiated chondrosarcoma (ex enchondroma in Ollier disease) or a metastatic carcinoma towards a pre-existing enchondroma. Histologically, it was consistent with metastatic squamous cell carcinoma, but the additional cartilaginous component was puzzling. Interestingly, scattered foci of hyaline cartilage were also in the metastatic carcinoma in a mixed growth pattern (Figs. 2c, d). Some of the cartilaginous lobules were surrounded by osteoid tissue. Pathologically, the cartilage components were most consistent with a pre-existing enchondroma in metastatic carcinoma. Immunohistochemical stains were performed. The tumour cells in the epithelial component were positive for AE1/AE3, CEA, CK5/6, P40 and CK7 and negative for TTF-1 and GATA3. Immunohistochemistry for IDH1 R132H antibody was performed in the tumours of the femur and the lung, and both were negative. The cartilaginous cells were weakly positive for IDH1 (Fig. 2e, f).

On a retrospective review of the MRI films using the criteria of Geirnaerdt et al. [2], the fat-suppressed T2-weighted images demonstrated three lesions in the left femur and tibia. The lesion in the anterior part of the medial condyle of the femur destroyed the cortical bone. It showed a slightly lower signal intensity than the other two lesions and was confirmed pathologically to be a metastasis. The other two lesions showed characteristic features of enchondroma. The lesion located in the lateral condyle showed blurry and irregular inner margin and adjacent bone oedema, which should be the co-existing tumour metastasis and enchondroma.

Imaging follow-up of the patient showed multiple metastatic tumours in the right 7th rib and bilateral frontal lobes of the brain (Figs. 1b, c). The patient expired approximately 1 year after the initial diagnosis.

## Discussion and literature review

Metastases are the most common type of malignancy involving the skeleton, and lung carcinoma is the second most common malignant tumour metastasizing to the bone both in women and men [3]. Metastasis of a lung carcinoma into a primary bone neoplasm is extremely rare, with only one case of metastasis of lung carcinoma to secondary chondrosarcoma reported previously [4].

Approximately 200 tumour-to-tumour metastasis cases have been reported since 1902 [5]. Berent first reported a case of metastatic squamous cell carcinoma from the jaw into renal cell carcinoma [6]. Fried in 1930 presented a case of bronchogenic carcinoma metastasizing into a meningioma [7]. Since then, various tumour-to-tumour metastasis case reports and series have been reported in the literature [8–12]. Based on the literature review, the most frequent metastatic donor is lung carcinoma, while renal cell carcinoma and meningioma are the most frequent malignant and benign recipients, respectively [5]. Campbell et al. [13] in 1968 reviewed previously published cases and proposed criteria to define tumour-to-tumour metastasis as follows: more than one distinctly separated primary tumours exist with the recipient tumour being a true neoplasm; the donor neoplasm must be a true metastasis with exclusion of tumour emboli or direct invasion from an adjacent tumour; exclude tumours that have metastasized to the lymphatic system.

In the present case, the first radiographic presentation of the patient was a pulmonary mass, which was pathologically diagnosed as a poorly differentiated squamous cell carcinoma of the lung. The follow-up MRI scan showed multiple lesions in the left femur and tibia, which were initially radiologically interpreted as metastasis, but pathologic examination revealed a diagnosis of enchondroma for left proximal tibial lesion and metastatic lung carcinoma and co-existent enchondroma for the distal left femoral lesion. Subsequent re-review of the MRI image confirmed that the left proximal tibial lesion and one of the lesions in the distal left femur were enchondromas, while the other distal femoral lesion was a malignant tumour partially “melting” with the enchondroma.

In this case, with the presence of both benign cartilaginous and high-grade malignant non-cartilaginous components, a differential diagnosis would be dedifferentiated chondrosarcoma. According to the latest WHO classification of bone tumours [14], the low-grade cartilaginous component can range from enchondroma-like appearance to grade 1 or grade 2 chondrosarcoma. The expression of keratin in the high-grade component cannot exclude dedifferentiated chondrosarcoma. Although the high-grade component almost always exhibits mesenchymal differentiation, it has been reported that rare cases of dedifferentiated chondrosarcoma can show epithelial differentiation, including squamous, glandular or adamantinoma-like features [15–19]. In such cases, detection of IDH1/2 gene mutations may be helpful in the diagnosis, which has been demonstrated in both high- grade and low-grade chondrosarcoma [20–22]. The positive IDH1 staining in the cartilaginous tumour and the negative staining in the poorly differentiated parts support the diagnosis of metastatic carcinoma as in dedifferentiated chondrosarcoma the IDH1 mutations persist [20].

Based on the literature, metastases to a benign tumour are more common than those to malignant tumours. A few factors have been proposed to explain the reason. The most reasonable one may be the benign tumour’s slow growth rate with low metabolic activity, which can provide prolonged exposure and a non-competitive environment for metastatic seeding and growth [23, 24]. These features are consistent with an enchondroma.

Bone metastases are common in practice, while the coexistence of metastases with benign or malignant primary bone neoplasms is rare in the literature. To our best knowledge, this is the first report of a lung carcinoma metastasizing to an enchondroma of a patient with Ollier disease. The diagnosis of metastatic carcinoma in a primary bone tumour can be challenging. It can be easily overlooked both radiologically and pathologically, which may lead to misdiagnosing a benign bone tumour as malignant tumour or mislabelling the metastatic carcinoma as dedifferentiated chondrosarcoma. Thorough clinical and radiological information is critical for an accurate diagnosis. Awareness of the tumour-to-tumour metastasis phenomenon by the pathologist can prevent inappropriate clinical intervention.
